# Field Studies Evaluating Bait Acceptance and Handling by Dogs in Navajo Nation, USA

**DOI:** 10.3390/tropicalmed2020017

**Published:** 2017-06-15

**Authors:** Scott Bender, David Bergman, Adrian Vos, Ashlee Martin, Richard Chipman

**Affiliations:** 1 Navajo Nation Veterinary Program, PO Box 2204, Chinle, AZ 86503, USA; scottbender@navajo-nsn.gov; 2 United States Department of Agriculture, Animal and Plant Health Inspection Service, Wildlife Services, 8836 N 23rd Avenue, Suite 2, Phoenix, AZ 85021, USA; david.l.bergman@aphis.usda.gov; 3 IDT Biologika GmbH, Am Pharmapark, Dessau-Rosslau 06861, Germany; ad.vos@idt-biologika.de; 4 United States Department of Agriculture, Animal and Plant Health Inspection Service, Wildlife Services, National Rabies Management Program, 59 Chenell Drive, Suite 2, Concord, NH 03301, USA; ashlee.d.martin@aphis.usda.gov

**Keywords:** rabies, bait, dog, oral vaccination

## Abstract

Mass parenteral vaccination remains the cornerstone of dog rabies control. Oral rabies vaccination (ORV) could increase vaccination coverage where free-roaming dogs represent a sizeable segment of the population at risk. ORV’s success is dependent on the acceptance of baits that release an efficacious vaccine into the oral cavity. A new egg-flavored bait was tested alongside boiled bovine intestine and a commercially available fishmeal bait using a hand-out model on the Navajo Nation, United States, during June 2016. A PVC capsule and biodegradable sachet were tested, and had no effect on bait acceptance. The intestine baits had the highest acceptance (91.9%; 95% confidence interval (CI), 83.9–96.7%), but the fishmeal (81.1%; 95% CI, 71.5–88.6%) and the egg-flavored baits (77.4%; 95% CI, 72.4–81.8%) were also well accepted, suggesting that local bait preference studies may be warranted to enhance ORV’s success in other areas where canine rabies is being managed. Based on a dyed water marker, the delivery of a placebo vaccine was best in the intestine baits (75.4%; 95% CI, 63.5–84.9%), followed by the egg-flavored (68.0%; 95% CI, 62.4–73.2%) and fishmeal (54.3%; 95% CI, 42.9–65.4%) baits. Acceptance was not influenced by the supervision or ownership, or sex, age, and body condition of the dogs. This study illustrates that a portion of a dog population may be orally vaccinated as a complement to parenteral vaccination to achieve the immune thresholds required to eliminate dog rabies.

## 1. Introduction

Dogs (*Canis lupus familiaris*) account for approximately 95% of all rabies cases reported worldwide, and they are also responsible for most human cases. Globally, the burden is not equal among countries [1]. Dog-mediated rabies has been eliminated from Europe, North America, and parts of Latin America. However, canine rabies remains a serious health problem in many countries in Africa and Asia. The most cost-effective method to control dog rabies is mass dog vaccination [2]. However, these campaigns have not always succeeded in achieving sufficient levels of herd immunity to interrupt the transmission cycle among dogs [3,4,5]. Poorly-supervised, free-roaming dogs play a pivotal role in transmission of rabies [6]. In some countries, a large proportion of the overall dog population is free-roaming, regardless of ownership. For example, over 50% and 66% of the owned dog population in Haiti and Bali, Indonesia, respectively, is allowed to roam freely [3,5]. During mass vaccination campaigns, these dogs are often not accessible for parenteral vaccination, or can only be vaccinated after considerable efforts to capture the free-roaming individuals [7,8]. Hence, alternative strategies—such as offering dogs vaccine baits—to reach these inaccessible dogs have been explored [9,10]. Initially, this novel approach targeted fox rabies in Europe and Canada. Later, this effective wildlife disease management tool was adapted for other reservoir species such as raccoon dogs (*Nyctereutes procyonoides*), raccoons (*Procyon lotor*), coyotes (*Canis latrans*), golden jackals (*Canis aureus*), and domestic dogs [11,12]. One of the essential components of this concept is a bait that is well accepted by the target population. Fishmeal baits used for the oral rabies vaccination (ORV) of red foxes (*Vulpes vulpes*) have been poorly accepted by dogs in many areas [13,14,15]. Nevertheless, a commercial fishmeal bait was accepted in previous studies on the Navajo Nation [16]. Several baits have been developed and tested in dogs around the world, including among others Mexico [17], Tunisia [18,19], Egypt [20], Turkey [13], the Philippines [21], and Guatemala [22]. Machine-made baits are generally not as well accepted as baits made from local available food sources [13,23]. However, the preparation of large quantities of baits from local food sources is problematic. Therefore, a well-accepted bait that can be produced on a large scale in a short period of time with quality assurance is required.

Most dogs on Navajo Nation lands are not subject to movement restrictions by their owners, and have little or no access to routine veterinary care, including vaccinations [16]. To react swiftly in the case of the re-emergence of rabies in the Navajo Nation, veterinary authorities have investigated the possibility of ORV in dogs, and bait studies have been previously conducted there [16,24,25]. The present study represents a continuation of these studies. The objective of this study was to evaluate the bait handling and acceptance of a recently developed bait by free-roaming dogs in the Navajo Nation. The new egg-flavored baits tested were previously found to be readily accepted by captive beagles and wolves (*Canis lupus*) [26].

## 2. Materials and Methods

### 2.1. Study Area

The Navajo Nation is a sovereign Native American Diné first nation located in the arid to semi-arid areas of high elevation (1600–3000 m) within northeastern Arizona, southeastern Utah, and northwestern New Mexico in the U.S. The reservation is 71,000 km².

**Figure 1:**
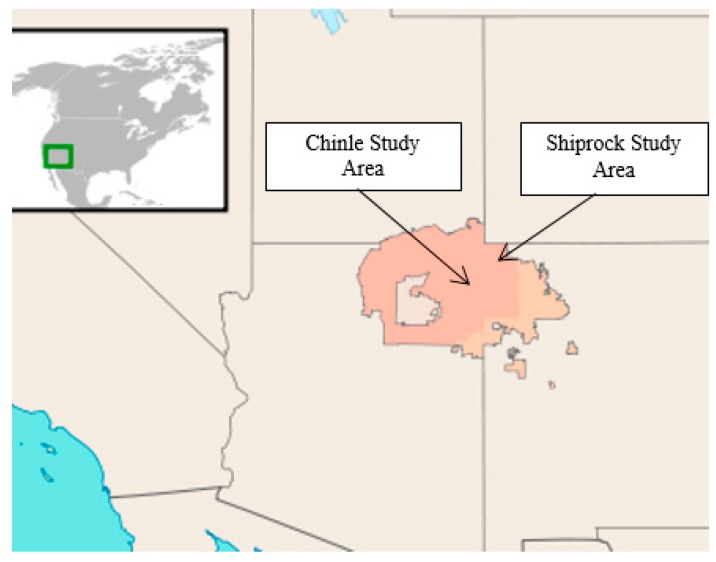
Location of the Navajo Nation (marked area) within the U.S., and the Chinle and Shiprock communities where the bait studies took place.

The Chinle (Chinle, Tsaile, Many Farms, Del Muerto, Lukachukai, Round Rock, and Rough Rock) and Shiprock (Shiprock, Hogback, and Cudii) Navajo Nation communities were selected for the bait trial, with the greater Chinle community located in Arizona, and the greater Shiprock community located in New Mexico (Figure 1). The study area comprised a combination of rural and semi-urban areas.

### 2.2. Field Trial

**Figure 2:**
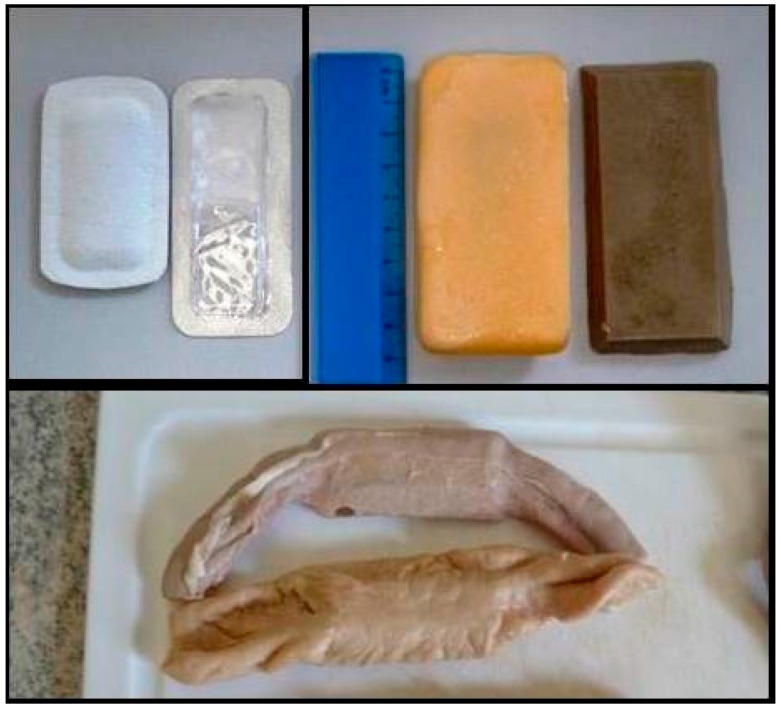
The two blister types used (left top; left—biodegradable, right —PVC) and the experimental egg-flavored bait (right top—yellow), fishmeal bait (right-top—brown), and intestine bait with capsule (bottom).

Three different baits and two different blister systems were tested (Table 1). All blisters contained dyed water and no active ingredients. As part of the overall field evaluation, the dye was used to visually aid field personnel to see if any of the liquid from the bait was in the mouths of the dogs as they ingested the bait. The dye was Patentblau V (Sigma Aldrich), a blue dye used in the food industry that is safe for human consumption and therefore was assumed to be safe for dogs. All three baits have recently been tested with captive beagles and European wolves [26] (Figure 2). In addition to a new bait comprised of gelatin and egg powder, a bait matrix of boiled section of cow intestine and a bait matrix identical to a commercially available bait made from vegetable fats and fishmeal used in ORV in red foxes was tested. One blister was a PVC capsule sealed by aluminum foil previously tested in Turkey and the Philippines [21,27] and the other was a sachet made from biodegradable foil and is still under final development.

Three person teams visited their allocated sections of the study area and systematically searched for dogs between 09:00 and 19:00 during 13–15 June 2016. If a dog was located, it was first determined if the dog had an owner and, if so, the dog was only offered a single bait with consent of the owner. Owners were given a leaflet that contained additional information and a phone number to report any potential adverse reactions. If the dog did not appear to have an owner, it was determined based on behavior if the dog was a stray/unowned (free-ranging dogs that survived and reproduced independently of human interaction or assistance) or community owned (free-ranging dogs that were fed and cared for by the community, but were not claimed by or residing with one single resident) by the ease of approachability of the dog. Community dogs were friendly and easily approached, while stray dogs were more cautious and would scare easily. All located dogs were offered a bait; stray/unowned and community dogs were approached and offered baits similar to owned dogs, with the exception that unowned dogs were approached more carefully to not scare them away. Bait type offered was randomly pre-determined. Half of all three bait types contained a hard PVC-capsule and the other half of all three bait types contained a soft biodegradable sachet. For every four experimental egg-flavored baits offered, one intestine and one fishmeal bait was included. The 4:1:1 ratio of bait flavor and the ratio of blister type were included in the randomization procedure. Since no data was available for bait uptake of the new egg-flavored population, this ratio were used to obtain as much data as possible on this bait. The intestine bait and fishmeal bait was used as a positive and negative control for comparison, respectively; it was assumed that the intestine bait would be well accepted and the fishmeal bait would be accepted poorly based on previous studies in the Philippines and Turkey [21,27]. A form was filled out during every baiting attempt to collect data on bait acceptance and handling. Bait handling describes what the dog does with the bait after acceptance, including handling time (time spent by the dog manipulating the bait; time was grouped into time intervals: <10 s, 10−30 s, 30−60 s, or >60 s) and if the vaccine container was perforated, discarded or swallowed. Based on these observations, an assessment was made by the observer if the dog would most likely have been successfully vaccinated based on the release of the contents of the blister into the oral cavity (“effectiveness”). Additional information recorded for each dog included: ownership, level of supervision, sex, age, size, and body condition. If the dog discarded the vaccine blister or if the dog did not accept the bait, the bait was recollected by the observer. 

Data were initially analyzed using the statistical software package Modde 10.0, Umetrics by fitting the data in a model by multilinear regression or partial least squares (PLS). However, all but one factor examined were qualitative (blister type, day, ownership, level of supervision, sex, age, size, and body condition). The responses measured (bait acceptance and vaccinated) were also qualitative (yes, no). Since qualitative factors tend to negatively influence the statistical power, the model had an extremely poor fit and most of the model coefficients were not significant; only bait type, intestine (positive), and fish meal (negative) had a significant effect. Therefore, data were analyzed with chi-squared using the statistical software package GraphPad Prism 6. 

## 3. Results

A total of 532 baiting attempts were recorded. Twenty-two data sheets with conflicting data were omitted from the analysis. Eighteen of the 22 omitted data sheets originated from a single team on the first day; most entries on those data sheets were not filled out or were filled out incorrectly. Also, two dogs ran off immediately when approached, and one dog was chased away by a passing car before it reached the bait offered. Hence, a total of 507 data sets was used for analysis, although it was not always possible to fill out the complete form. If no data was available for a specific parameter investigated, the data set was not included in the analysis for that parameter. Additional analysis on data collected for the various parameters of this study are included as Supplementary Tables S1–S9. 

**Figure 3:**
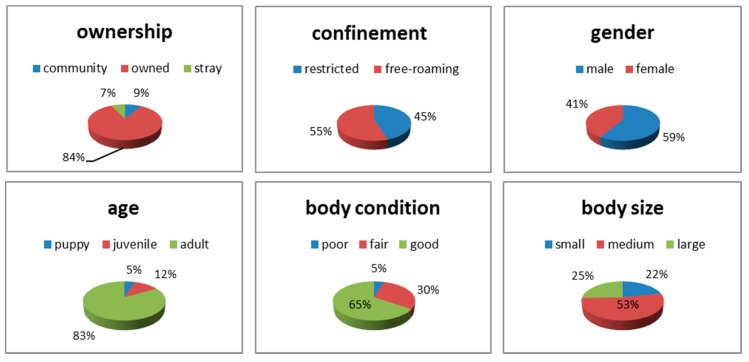
Characteristics of the dogs included in this study.

**Figure 4:**
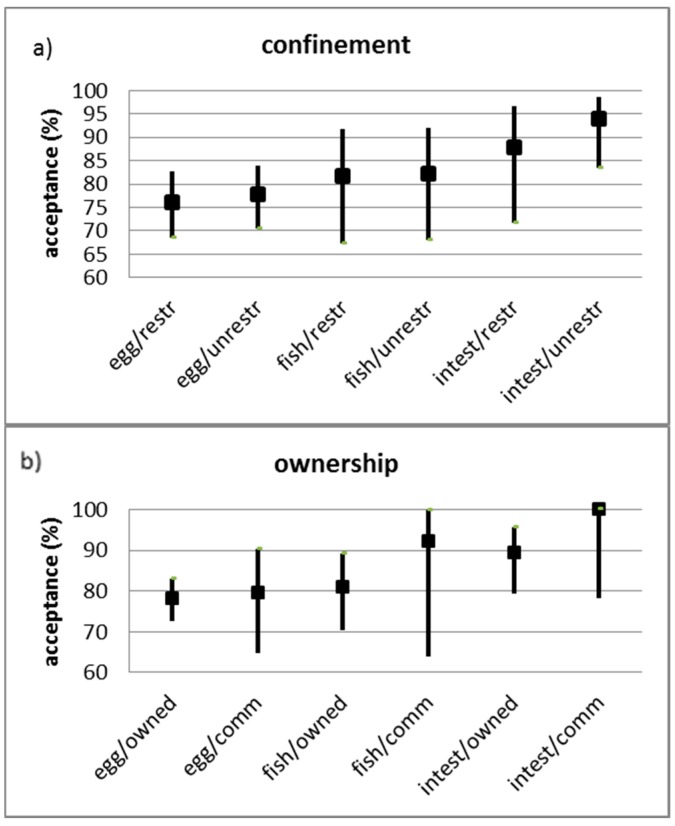
Bait acceptance (%) (mean percentage and 95% confidence interval) associated with (**a**) level of confinement (restr—restricted; unrest—unrestricted) and (**b**) ownership status (comm—community).

The ownership, confinement status, gender, age classification, body condition, and body size for the target dog population was documented and summarized (Figure 3). There were no significant effects on bait acceptance associated with dog age, sex, body condition, and size by contingency analysis. The overall bait acceptance (consumption) was 80.6% (95% CI, 76.8–83.9%; Table 2); the intestine baits were refused significantly less often than the other two baits (χ² = 9.091, df = 2, *p* = 0.01). The type of blister had no significant effect on bait acceptance (χ² = 1.276, df = 1, *p* = 0.26). The dogs’ level of confinement (restricted vs. unrestricted) or ownership status (owned vs. community, including ownerless) had no significant effect on bait acceptance (Figure 4a,b). Also, no significant effect in bait acceptance was observed for the time of day that baits were offered to the dogs (χ² = 3.765, df = 4, *p* = 0.44).

**Figure 5:**
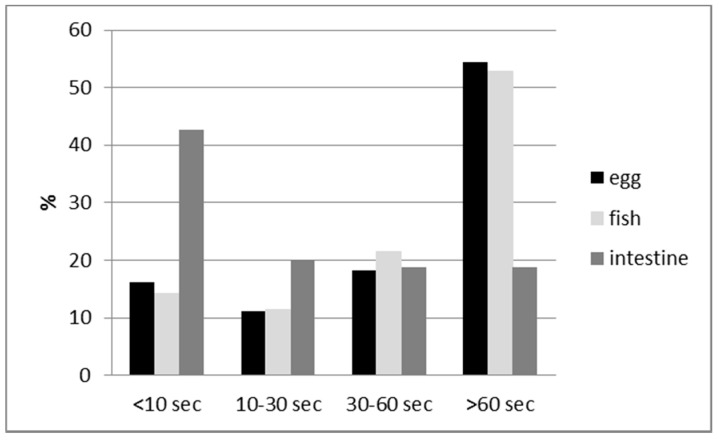
Bait handling time (seconds) for the 3 different baits.

When baits were accepted by the dogs, 90.1% (95% CI, 86.6–93.0%) of the animals consumed the whole bait. There was no significant difference between blisters (χ² = 0.8826, df = 1, *p* = 0.35) and bait type (χ² = 5.213, df = 2, *p* = 0.07) if the bait offered was consumed completely or partially. The intestine baits were consumed significantly faster than the other two baits (χ² = 40.90, df = 6, *p* < 0.0001) (Figure 5). The baits containing a sachet were also consumed significantly more rapidly than baits with the hard PVC capsule (χ² = 10.65, df = 3, *p* = 0.01). The intestine and sachet combination was consumed significantly much faster in comparison to intestine baits with the capsule; 59% (95% CI, 42.1–74.4%) of the intestine baits accepted were consumed within 10 s (χ² = 10.95, df = 3, *p* = 0.01). No significant difference in consumption time between the blister types was observed for the egg baits (χ² = 1.413, df = 3,s *p* = 0.70) and fishmeal baits (χ² = 4.689, df = 3, *p* = 0.20).

There was a significant effect on the fate of the blister (swallowed or discarded) for both the bait and blister type. Almost all dogs (84.2%; 95% CI, 74.0–91.6%) that consumed an intestine bait swallowed the blister, while only 58.6% (95% CI, 52.1–61.0%) and 38.2% (95% CI, 26.7–50.8%) of the dogs that accepted the egg and fishmeal baits, respectively, swallowed the blister (χ² = 32.19, df = 2, *p* < 0.0001). Irrespective of bait type, the sachets were swallowed more often than the PVC capsules; 81.3% (95% CI, 74.6–86.8%) of dogs swallowed the sachet, but only 42.9% (95% CI, 30.1–49.8%) of the capsules were swallowed (χ² = 58.05, df = 1, *p* < 0.0001). However, overall, the type of blister did not have an influence on bait handling time.

The blisters in the egg baits (94.8%; 95% CI, 90.9–97.4%) were more often perforated than those in the fishmeal baits (82.3%; 95% CI, 70.5–90.8%) and intestine baits (87.0%; 95% CI, 75.1–94.6%; χ² = 10.83, df = 2, *p* = 0.004). Blister type did not seem to have any influence on perforation, except for the intestine baits, in which the PVC capsules (96.6%; 95% CI, 82.2–99.9%) were significantly perforated more often than the sachets (76.0%; 95% CI, 54.9–90.6%; χ² = 5.026, df = 1, *p* = 0.03).

Overall, there was no significant effect of blister type on vaccination success based on dyed water marker (χ² = 0.3145, df = 1, *p* = 0.5749), but there was a highly significant effect of bait type on vaccination success (effectiveness) (χ² = 17.52, df = 2, *p* = 0.0002). The fishmeal baits had a significantly lower vaccine delivery success rate than the other two baits. The content of the blister was considered successfully released in the oral cavity of 68.8% (95% CI, 55.9–79.8%) of the dogs that accepted the fishmeal baits. For the egg and intestine baits, 89.9% (95% CI, 85.2–93.5%) and 83.9% (95% CI, 72.3–92.0%) of the dogs were considered successfully ‘vaccinated’, respectively. The better accepted intestine baits had a lower vaccination success rate due to the very short handling time; dogs that consumed baits in less than 10 s were significantly more often considered ‘not vaccinated’ (χ² = 33.45, df = 3, *p* < 0.0001). The low success rate of the fishmeal baits was predominantly caused by the blister type; using the hard PVC capsule, only 55.6% (95% CI, 38.1–72.1%) of the dogs that accepted the bait were considered vaccinated, while for the sachet this percentage was 85.7% (95% CI, 67.3–96.0%; χ² = 6.668, df = 1, *p* = 0.01). For the other two baits, no significant effect of the blister was detected. A total of 91.2% (95% CI, 84.8–95.5%) and 88.2% (95% CI, 80.4–93.8%) of the dogs accepting the egg baits containing the capsule or the sachet, respectively, were considered vaccinated (χ² = 0.54, df = 1, *p* = 0.46).

**Figure 6:**
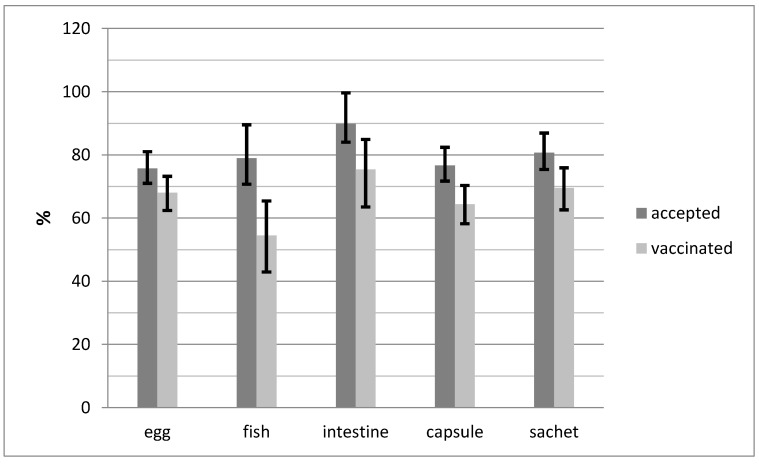
The difference in the proportion of dogs accepting and consuming baits and blisters and the subsequent assessment of a successful vaccination attempt (release of contents of vaccine blister in oral cavity) with associated 95% confidence intervals.

A total of 87.9% (95% CI, 71.8–96.6%) and 79.3% (95% CI, 60.3–92.0%) of the dogs accepting the intestine baits with the capsule and sachet, respectively, were considered vaccinated (χ² = 0.84, df = 1, *p* = 0.36). If we analyze bait acceptance and vaccination success in tandem, the discrepancy between acceptance and vaccination is much lower for the egg baits than for the other two baits (Figure 6). The effectiveness of the fishmeal baits was much lower than the other two bait types.

## 4. Discussion

An important precondition for oral vaccination is the availability of a bait that is well accepted by the target species under field conditions. Initially, efforts to develop baits for dogs were based on those previously developed for wildlife such as foxes and raccoons; a review of some of these early studies can be found in Linhart [28]. A bait that is poorly accepted by dogs has no use for oral vaccination of these animals against rabies, even if all of the other requirements have been fulfilled. Bait acceptance studies using a variety of baits have been conducted in different countries, and showed regional differences in bait preference [13,17,18,19,20,21,22]. For example, the significantly better accepted boiled intestine baits used in this study were only taken by 58.8% of the dogs in Guatemala. Poultry flavored baits were accepted more often than other bait types in Guatemala [22]. No significant difference in bait acceptance was observed between the fishmeal and egg-flavored baits in the Navajo Nation. During a previous study conducted on the Navajo Nation with confined shelter dogs, five flavors were tested using a commercial bait matrix: bacon, cheese, sweet, egg, and fish. The sweet-flavored baits were the least preferred, and among the other flavors no significant difference could be observed [25]. Interestingly, the acceptance of the egg-flavored baits (79.9%) was quite similar to that observed in the current study (77.4%), although a different ground bait substance was used. In another study at the dog shelter in the Navajo Nation, seven different flavors added to a commercial bait matrix were tested using the two-food-preference test approach [24]. Here, a dog-food flavor had the highest preference. However, the preference for dog-food flavor may have been a result of the familiarity of the dogs with this food source in the shelter. Bergman et al. [16] tested four different commercially available bait matrices, and found that the bait acceptance of free-roaming dogs in the Navajo Nation fluctuated between 56.5% and 84.5%. The fish-flavored coated sachet had the highest bait consumption among the four baits tested. The relatively high acceptance rate of the fishmeal baits in these previous studies and our study in Navajo Nation was rather unexpected, considering that fish is an unusual food source for these animals and the poor results obtained with fish-flavored baits in free-roaming and confined dogs in other areas [13,14,26]. However, in Sri Lanka, a commercially available fish-flavored bait was well accepted (84%) by owned dogs [29]. In several studies, a low acceptance of manufactured baits by free-roaming dogs was explained by the lack of familiarity with the taste, smell, shape, and texture of the baits [13]. Free-roaming dogs are often fed on household leftovers or offal. Hence, it can be expected that baits based on these resources are more attractive [30]. Thus, baits with a high acceptance rate in one area can be refused by dogs in another area, due to different local food preferences and experiences. Although several studies indicate high acceptance rates for manufactured baits [15,23,29], generally, baits made from locally available material have a much higher acceptance rate [13,21,22]. During a field study in Turkey, 96% of all dogs accepted the locally-made bait based on minced-meat mixed with bread crumbs [13]. In the Philippines, more than 90% of the dogs accepted baits made from different local materials [21]. Locally produced baits are often not favored by field personnel due to difficulties in preparing, handling, and storing the baits [22]. However, due to the observed regional differences in bait acceptance, it may be difficult to develop a universally well-accepted bait for dogs. Furthermore, dog rabies is predominantly a problem in developing countries with limited financial resources available for rabies control. The cost of purchasing imported manufactured vaccine-loaded baits (including costs for their transportation and cold-storage) will most likely exceed the cost of the importation of vaccine-loaded blisters that could be incorporated locally in a suitable bait [21].

As shown in this study, when a dog accepts a bait, it does not automatically mean that the animal will be successfully vaccinated. The investigational phase and bait handling period upon acceptance should not last too long, because it increases the risk that external factors will lead to an interruption, and, consequently, a failed vaccination attempt. Hence, the selected bait should be immediately attractive to the dogs [13]. The handling time of the intestine bait was significantly shorter than for the other two bait types. However, the animal should not swallow the bait immediately, including the vaccine blister; the vaccine needs to be released in the oral cavity, and, therefore, the blister has to be punctured before it is swallowed or discarded. Actually, it was shown that the dogs consuming the bait within ten seconds were significantly more often considered ‘not vaccinated’. The biodegradable sachet was significantly more often swallowed than the PVC capsule, but the type of blister did not influence the handling time of the bait. Dogs may separate the blister from the bait matrix, or it may drop out of the dogs’ mouth before being punctured due to poor adherence with the bait matrix. In this study, it was observed that the fishmeal baits often broke into pieces when tossed to the dogs, consequently separating the vaccine blister from the bait matrix. Even when the blister is punctured, it does not guarantee a successful vaccination; sometimes large amounts of the vaccine are retained within the blister, and the released amount would not be sufficient to induce a protective immune response. Finally, during bait handling, the vaccine virus released from the punctured blister can be spilled on the ground [21]. Hence, not only does bait palatability need to be investigated and optimized, but also the influence of the shape, size, and texture of the bait matrix, as well as the blister, needs careful consideration in terms of the efficient release of the vaccine in the oral cavity. Experimental studies where the uptake of a single vaccine bait protected the dogs against a subsequent challenge infection have shown that this is feasible [27,31].

The swallowing of the PVC capsule can cause an adverse reaction (gastric intolerance) in dogs when swallowed [32], although no such adverse events were reported after the present study. The biodegradable sachet will cause less intolerance when swallowed. The swallowing of the blister after prolonged chewing actually has an advantage, by reducing the amount of discarded blisters in the environment. At the moment, it is suggested to re-collect all of the discarded vaccine containers to reduce unintentional human contact with the vaccine virus. However, sometimes the dog does not consume the bait immediately, but walks away with it, and subsequently the vaccine blister cannot be re-collected. If a vaccine container was used that is frequently swallowed, the number of discarded blisters not re-collected would be further reduced.

This study found no significant effect of the dogs’ age, sex, body condition, and size on bait acceptance. Therefore, the age distribution, gender ratio, or health of a local dog population is not expected to impact the bait acceptance or the vaccination success of a population when using the bait types assessed in this study. There was also no significant effect on bait acceptance associated with ownership status or the dogs’ level of confinement. Thus, similar conclusions could be drawn on the population regardless of the ownership status and level of confinement of the dogs.

It can be concluded that both bait types, manufactured or made from local material, were well accepted by the local dog population, but the bait, the blister, and the interaction between these two clearly influenced bait handling, and consequently the outcome of the vaccination attempt. There is a discrepancy between the bait acceptance rate and the subsequent estimated vaccination rate. It seems that the difference between these two was the lowest for the newly developed egg-flavored baits. However, this bait can be further optimized, and potentially this discrepancy could be further reduced. It was shown that several dogs had problems with the shape and texture of this bait. The animals had problems picking up the bait from the ground (a condition of shape) and sometimes seemed to consider the bait more as a toy than as a food item (a condition of texture). Both aspects increased the handling time, and subsequently the risk of disturbance and discontinuation of bait consumption. With minor improvements to the bait and further research, the egg-flavored baits have potential to be effectively used as an ORV bait for dogs in the Navajo Nation.

## Figures and Tables

**Table 1:** Bait and blister components making up the combinations field tested on Navajo Nation, June 2016.

	Material	Size (cm)	Weight (gr)
**Bait**			
Intestine	boiled local cow intestine sections	8–12 cm long	20–30
Fishmeal	vegetable fats + fishmeal	8.5 × 4.0 × 1.2	43
Egg	gelatin + egg powder	8.5 × 4.0 × 1.2	43
**Blister**			
Capsule	PVC + aluminum foil	6.5 × 3.0 × 0.7	3.5 mL
Sachet	Biodegradable foil	5.0 × 3.0 × 0.4	3.5 mL

**Table 2:** Summary of bait acceptance and handling, fate of blister and final assessment if vaccine was released in oral cavity of the dog (vaccinated), included 95% confidence intervals (95% CI). Except for bait acceptance all numbers are based on dogs that (partially) consumed the bait after it was accepted.

		Bait Accepted			Bait Consumed Completely			Blister Swallowed			Blister Perforated			‘Vaccinated’		
**Bait**	**Blister**	**n**	**%**	**95% CI**	**n**	**%**	**95% CI**	**n**	**%**	**95% CI**	**N**	**%**	**95% CI**	**N**	**%**	**95% CI**
egg	capsule	140	74.5	69.3–82.1	106	86.9	79.6–92.3	57	42.2	33.8–51.0	112	94.9	89.3–98.1	114	91.2	84.8–95.5
	sachet	110	81.5	73.9–87.6	90	90.0	82.4–95.1	82	80.4	71.4–87.6	90	94.7	88.1–98.3	90	88.2	80.4–93.8
fish	capsule	40	87.0	73.7–95.1	34	87.2	72.6–95.7	6	15.8	6.0–31.3	29	78.4	61.8–90.2	20	55.6	38.1–72.1
	sachet	33	75.0	59.7–86.8	28	90.3	74.2–98.0	20	66.7	47.2–82.7	22	88.0	68.8–97.5	24	85.7	67.3–96.0
intestine	capsule	39	88.6	75.4–96.2	34	97.1	75.2–97.1	27	73.0	55.9–86.2	28	96.6	82.2–99.9	29	87.9	71.8–96.6
	sachet	40	95.2	83.8–99.4	37	97.4	86.2–99.9	37	94.9	82.7–99.4	19	76.0	54.9–90.6	23	79.3	60.3–92.0
**Total**		402	80.6	76.8–83.9	329	90.1	86.6–93.0	229	60.3	55.0–65.1	300	91.2	87.6–94.0	300	85.0	80.8–88.5

## Supplementary Materials

The following are available online at http://www.mdpi.com/2414-6366/2/2/17/s1, Table S1. Bait acceptance for the 3 bait types and 2 blister types; unknowns not further included in statistical data analysis, Table S2. Amount (%) of bait consumed by dog upon acceptance for the 3 bait types and 2 blister types; unknowns not further included in statistical data analysis, Table S3. Bait handling time (seconds) upon acceptance for the 3 bait types and 2 blister types; unknowns not further included in statistical data analysis, Table S4. The number of animals that swallowed or discarded the blister after bait acceptance for the 3 bait types and 2 blister types; unknowns not further included in statistical data analysis, Table S5. Number of blisters perforated/ruptured (yes) or not (no) after bait acceptance for the 3 bait types and 2 blister types; unknowns not further included in statistical data analysis, Table S6. Number of dogs that accepted a bait and were considered vaccinated (likely) or not (not likely) in case that the blister would have contained a vaccine for the 3 bait types and 2 blister types; unknowns not further included in statistical data analysis, Table S7. Bait acceptance and the effectiveness (dog likely to be vaccinated) for the 3 bait types and 2 blister types. Only animals were included for which data on both parameters, acceptance and effectiveness (likely vaccinated), were available, Table S8. Bait handling time (seconds) and the subsequent effectiveness of the vaccination attempt (likely or not likely vaccinated) for the 3 bait types; unknowns (effectiveness) not included in data analysis, and Table S9. Bait acceptance by owned and community (including ownerless) dogs for the 3 bait types and 2 blister types; unknowns (ownership status) not included in data analysis.
