# Supplementary material for: Field Studies Evaluating Bait Acceptance and Handling by Dogs in Navajo Nation, USA
Source: Trop Med Infect Dis. 2017 Jun 15;2(2):17. doi: 10.3390/tropicalmed2020017 (PMC6082073; doi:10.3390/tropicalmed2020017)
Supplement: Supplementary file 1 [file tropicalmed-02-00017-s001.pdf]

Article

Field studies evaluating bait acceptance and handling by dogs in Navajo Nation, USA

Scott Bender [1], David Bergman [2], Ad Vos [3], Ashlee Martin[4], Richard Chipman [4,*]

[1] Navajo Nation Veterinary Program, USA; scottbender@navajo-nsn.gov
[2] USDA, APHIS, WS, Arizona Wildlife Services, USA; David.L.Bergman@aphis.usda.gov
[3] IDT Biologika GmbH, GERMANY; Ad.Vos@idt-biologika.de
[4] USDA, APHIS, WS, National Rabies Management Program, USA; Ashlee.D.Martin@aphis.usda.gov
* Corresponding Author: Richard.B.Chipman@aphis.usda.gov; Tel.: 603-223-9623

Supplemental Material:

Table S1. Bait acceptance for the 3 bait types and 2 blister types; unknowns not further included in statistical data analysis.

		Yes			No			Unknown
**Bait**	**Blister**	**n**	**%**	**95% CI**	**n**	**%**	**95%CI**	**n**
**Egg**	Hard	140	74.5	67.6 – 80.5	48	25.5	19.5 – 32.4	4
	Soft	110	81.5	73.9 – 87.6	25	18.5	12.4 – 26.1	3
	Subtotal	***250***	***77.4***	***72.4 – 81.8***	***73***	***22.6***	***18.2 – 27.6***	***7***
**Fish**	Hard	40	87.0	73.7 – 95.1	6	13.0	4.9 – 26.3	-
	Soft	33	75.0	59.7 – 86.8	11	25.0	13.2 – 40.3	1
	Subtotal	***73***	***81.1***	***71.5 – 88.6***	***17***	***23.3***	***11.4 – 28.5***	***1***
**Intestine**	Hard	39	88.6	75.4 – 96.2	5	11.4	3.8 – 24.6	-
	Soft	40	95.2	83.8 – 99.4	2	4.8	0.6 – 16.2	-
	Subtotal	***79***	***91.9***	***83.9 – 96.7***	***7***	***8.1***	***3.3 – 16.1***	***-***
**Subtotal**	Hard	219	80.0	73.5 – 83.4	59	20.0	16.6 - 26.5	4
	Soft	183	82.8	77.2 – 87.5	38	17.2	12.5 – 22.8	4
**Total**		**402**	**80.6**	**76.8 – 83.9**	**97**	**19.4**	**16.1 – 23.2**	**8**

Table S2. Amount (%) of bait consumed by dog upon acceptance for the 3 bait types and 2 blister types; unknowns not further included in statistical data analysis.

		<50%			>50%			100%			Unknown
**Bait**	**Blister**	**n**	**%**	**95% CI**	**n**	**%**	**95% CI**	**n**	**%**	**95% CI**	**n**
**Egg**	Hard	11	9.0	4.6 – 15.6	5	4.1	1.3 – 9.3	106	86.9	79.6 – 92.3	18
	Soft	3	3.0	0.6 – 8.5	7	7.0	2.9 – 13.9	90	90.0	82.4 – 95.3	10
	Subtotal	***14***	***6.3***	***3.5 – 10.4***	***12***	***5.4***	***2.8 – 9.3***	***196***	***88.3***	***83.3 – 92.2***	***28***
**Fish**	Hard	2	5.1	0.6 – 17.3	3	7.7	1.6 – 20.9	34	87.2	72.6 – 95.7	1
	Soft	1	3.2	0.1 – 16.7	2	6.5	0.8 – 21.4	28	90.3	74.2 – 98.0	2
	Subtotal	***3***	***4.3***	***0.9 – 12.0***	***5***	***7.1***	***2.4 – 15.9***	***62***	***88.6***	***78.7 – 94.9***	***3***
**Intestine**	Hard	-	-	0 – 10.0	1	2.9	0.1 – 14.9	34	97.1	85.1 – 99.9	4
	Soft	-	-	0 – 9.3	1	2.6	0.1 – 13.8	37	97.4	86.2 – 99.9	2
	Subtotal	***-***	***-***	***0 – 4.9***	***2***	***2.7***	***0.3 – 9.5***	***71***	***97.3***	***90.5 – 99.7***	***6***
**Subtotal**	Hard	13	6.6	3.6 – 11.1	9	4.6	2.1 – 8.5	174	88.8	83.5 – 92.8	23
	Soft	4	2.4	0.6 – 5.9	10	5.9	2.9 – 10.6	155	91.7	86.5 – 95.4	14
**Total**		**17**	**4.7**	**2.7 – 7.4**	**19**	**5.2**	**3.2 – 8.0**	**329**	**90.1**	**86.6 - 93.0**	**37**

Table S3. Bait handling time (seconds) upon acceptance for the 3 bait types and 2 blister types; unknowns not further included in statistical data analysis.

		<10 sec			10-30 sec			30-60 sec			>60 sec			Unknown
**Bait**	**Blister**	**n**	**%**	**95% CI**	**n**	**%**	**95% CI**	**n**	**%**	**95% CI**	**n**	**%**	**95% CI**	**n**
**Egg**	Hard	19	14.5	9.0 – 21.7	13	9.9	5.4 – 16.4	24	18.3	12.1 – 26.0	75	57.3	48.3 – 65.9	9
	Soft	19	17.8	11.0 – 26.3	14	13.1	7.3 – 21.0	20	18.7	11.8 – 27.4	54	50.5	40.6 – 60.3	3
	Subtotal	***38***	***16.0***	***11.6 – 21.3***	***27***	***11.3***	***7.6 – 16.1***	***44***	***18.5***	***13.8 – 24.0***	***129***	***54.2***	***47.6 – 60.7***	***12***
**Fish**	Hard	3	7.7	1.6 – 20.9	4	10.3	2.9 – 24.2	11	28.2	15.0 – 44.9	21	53.8	37.2 – 69.9	1
	Soft	7	22.6	9.6 – 41.1	4	12.9	3.6 – 29.8	4	12.9	3.6 – 29.8	16	51.6	33.1 – 69.8	2
	Subtotal	***10***	***14.3***	*7.1 – 24.7*	***8***	***11.4***	*5.1 – 21.3*	***15***	***21.4***	*12.5 - 32.9*	***37***	***52.9***	*40.6 – 64.9*	***3***
**Intestine**	Hard	9	25.0	12.1 - 42.2	8	22.2	10.1 – 39.2	8	22.2	10.1 – 39.2	11	30.6	16.3 – 48.1	3
	Soft	23	59.0	42.1 – 74.4	7	17.9	7.5 – 33.5	6	15.4	5.9 – 30.5	3	7.7	1.6 – 20.9	1
	Subtotal	***32***	***42.7***	*31.3 – 54.6*	***15***	***20.0***	*11.6 – 30.8*	***14***	***18.7***	*10.6 – 29.3*	***14***	***18.7***	*10.6 – 29.3*	***4***
**Subtotal**	Hard	31	15.0	10.5 – 20.7	25	12.1	8.0 – 17.4	43	20.9	15.5 – 27.1	107	51.9	44.9 – 58.9	13
	Soft	49	27.7	21.2 – 34.9	25	14.1	9.4 – 20.1	30	16.9	11.7 – 23.3	73	41.2	33.9 – 48.9	6
**Total**		**80**	**20.9**	16.9 – 25.3	**50**	**13.1**	9.8 – 16.8	**73**	**19.1**	15.2 – 23.4	**180**	**47.0**	41.9 – 52.1	**19**

Table S4. The number of animals that swallowed or discarded the blister after bait acceptance for the 3 bait types and 2 blister types; unknowns not further included in statistical data analysis.

		Swallowed			Discarded			Unknown
**Bait**	**Blister**	**n**	**%**	**95% CI**	**n**	**%**	**95% CI**	**n**
**Egg**	Hard	57	42.2	33.8 – 51.0	78	57.8	49.0 – 66.2	5
	Soft	82	80.4	71.4 – 87.6	20	19.6	12.4 – 28.6	8
	Subtotal	***139***	***58.6***	***52.1 – 65.0***	***98***	***41.4***	***35.0 – 47.9***	***13***
**Fish**	Hard	6	15.8	6.0 – 31.3	32	84.2	68.7 – 94.0	2
	Soft	20	66.7	47.2 – 82.7	10	33.3	17.3 – 52.8	3
	Subtotal	***26***	***38.2***	***26.7 – 50.8***	***42***	***61.8***	***49.2 – 73.3***	***5***
**Intestine**	Hard	27	73.0	55.9 – 86.2	10	27.0	13.8 – 44.1	2
	Soft	37	94.9	82.7 – 99.4	2	5.1	0.6 – 17.3	1
	Subtotal	***64***	***84.2***	***74.0 – 91.6***	***12***	***15.8***	***8.4 – 26.0***	***3***
**Subtotal**	Hard	90	42.9	30.1 – 49.8	120	57.1	50.2 – 63.9	9
	Soft	139	81.3	74.6 – 86.8	32	18.7	13.2 – 25.4	12
**Total**		**229**	**60.3**	**54.0 – 65.1**	**152**	**39.9**	**34.9 – 45.0**	**21**

Table S5. Number of blisters perforated / ruptured (yes) or not (no) after bait acceptance for the 3 bait types and 2 blister types; unknowns not further included in statistical data analysis.

**Blister Perforated:**		Yes			No			Unknown
**Bait**	**Blister**	**n**	**%**	**95% CI**	**n**	**%**	**95% CI**	**n**
**Egg**	Hard	112	94.9	89.3 – 98.1	6	5.1	1.9 – 10.7	22
	Soft	90	94.7	88.1 – 98.3	5	5.3	1.7 – 11.9	15
	Subtotal	***202***	***94.8***	***90.9 – 97.4***	***11***	***5.2***	***2.6 – 9.1***	***37***
**Fish**	Hard	29	78.4	61.8 – 90.2	8	21.6	9.8 – 38.2	3
	Soft	22	88.0	68.8 – 97.5	3	12.0	2.5 – 31.2	8
	Subtotal	***51***	***82.3***	***70.5 – 90.8***	***11***	***17.7***	***9.2 – 29.5***	***11***
**Intestine**	Hard	28	96.6	82.2 – 99.9	1	3.4	0.1 – 17.8	10
	Soft	19	76.0	54.9 – 90.6	6	24.0	9.4 – 45.1	15
	Subtotal	***47***	***87.0***	***75.1 – 94.6***	***7***	***13.0***	***5.4 – 24.9***	***25***
**Subtotal**	Hard	169	91.8	86.9 – 95.4	15	8.2	4.6 – 13.1	35
	Soft	131	90.3	84.3 – 94.6	14	9.7	5.4 – 15.7	38
**Total**		**300**	**91.2**	**87.6 – 94.0**	**29**	**8.8**	**6.0 – 12.4**	**73**

Table S6. Number of dogs that accepted a bait and were considered vaccinated (likely) or not (not likely) in case that the blister would have contained a vaccine for the 3 bait types and 2 blister types; unknowns not further included in statistical data analysis.

**Vaccination:**		**Likely**			**Not Likely**			**Unknown**
**Bait**	**Blister**	**n**	**%**	**95% CI**	**n**	**%**	**95% CI**	**n**
**Egg**	Hard	114	91.2	84.8 - 95.5	11	8.8	4.5 – 15.2	15
	Soft	90	88.2	80.4 – 93.8	12	11.8	6.2 – 19.6	8
	Subtotal	***204***	***89.9***	***85.2 – 93.5***	***23***	***10.1***	***6.5 – 14.8***	***23***
**Fish**	Hard	20	55.6	38.1 – 72.1	16	44.4	27.9 – 61.9	4
	Soft	24	85.7	67.3 – 96.0	4	14.3	4.0 – 32.7	5
	Subtotal	***44***	***68.8***	***55.9 – 79.8***	***20***	***31.3***	***20.2 – 44.1***	***9***
**Intestine**	Hard	29	87.9	71.8 – 96.6	4	12.1	3.4 – 28.2	6
	Soft	23	79.3	60.3 – 92.0	6	20.7	8.0 – 39.7	11
	Subtotal	***52***	***83.9***	***72.3 – 92.0***	***10***	***16.1***	***8.0 – 21.9***	***17***
**Subtotal**	Hard	163	84.0	78.1 – 88.9	31	16.0	11.1 – 21.9	25
	Soft	137	86.2	79.8 – 91.1	22	13.8	8.9 – 20.2	24
**Total**		**300**	**85.0**	**80.8 – 88.5**	**53**	**15.0**	**11.5 – 19.2**	**49**

Table S7. Bait acceptance and the effectiveness (likelihood of vaccination) for the 3 bait types and 2 blister types analyzed from animals for which data on both parameters (acceptance and effectiveness), were available (N = 450). This included individuals that did not accept the bait and therefore were not vaccinated.

		**N**	**Bait Accepted**			**Effectiveness**		
**Bait**	**Blister**		**n**	**%**	**95% CI**	**n**	**%**	**95% CI**
**Egg**	Hard	173	125	72.3	64.9 – 78.8	114	65.9	58.3 – 72.9
	Soft	127	102	80.3	72.3 – 86.8	90	70.9	62.1 – 78.6
	Subtotal	***300***	***227***	***75.7***	***70.4 – 80.4***	***204***	***68.0***	***62.4 – 73.2***
**Fish**	Hard	42	36	85.7	71.5 – 94.6	20	47.6	32.0 – 63.6
	Soft	39	28	71.8	55.1 – 85.0	24	61.5	44.6 – 76.6
	Subtotal	***81***	***64***	***79.0***	***68.5 – 87.3***	***44***	***54.5***	***42.9 – 65.4***
**Intestine**	Hard	38	33	86.8	71.9 – 95.6	29	76.3	59.8 – 88.6
	Soft	31	29	93.5	78.6 – 99.2	23	74.2	55.4 – 88.1
	Subtotal	***69***	***62***	***89.9***	***80.2 – 95.8***	***52***	***75.4***	***63.5 – 84.9***
**Subtotal**	Hard	253	194	76.7	71.0 – 81.7	163	64.4	58.2 – 70.3
	Soft	197	159	80.7	74.5 – 86.0	137	69.5	62.6 – 75.9
**Total**		**450**	**353**	**78.4**	**74.4 – 82.2**	**300**	**66.7**	**62.1 – 71.0**

Table S8. Bait handling time (seconds) and the subsequent effectiveness of the vaccination attempt (likely or not likely vaccinated) for the 3 bait types; unknowns (effectiveness) not included in data analysis.

Handling Time (sec)	Bait	Likely			No		
		n	%	95% CI	n	%	95% CI
<10	Egg	16	55.2	35.7 – 73.6	13	44.8	26.4 – 64.3
	Fish	3	50.0	11.8 – 88.2	3	50.0	11.8 – 88.2
	Intestine	13	65.0	40.8 – 84.6	7	35.0	15.4 – 59.2
	Total	***32***	***58.2***	***44.1 – 71.3***	***23***	***41.8***	***55.9 – 28.7***
10-30	Egg	24	92.3	74.9 – 99.1	2	7.7	0.9 – 25.1
	Fish	6	75.0	34.9 – 96.8	2	25.0	3.2 – 65.1
	Intestine	11	91.7	61.5 – 99.8	1	8.3	0.2 – 38.5
	Total	***41***	***89.1***	***76.4 – 96.4***	***5***	***10.9***	***3.6 – 23.6***
30-60	Egg	42	100.0	91.6 – 100	0	0.0	0 - 8.4
	Fish	9	60.0	32.3 – 83.7	6	40.0	16.3 – 67.7
	Intestine	13	92.9	66.1 – 99.8	1	7.1	0.2 – 33.9
	Total	***64***	***90.1***	***80.7 – 95.9***	***7***	***9.9***	***4.1 – 19.3***
>60	Egg	111	93.3	87.2 – 97.1	8	6.7	2.9 – 12.8
	Fish	23	71.9	53.3 – 86.3	9	28.1	13.7 – 46.7
	Intestine	11	91.7	61.5 – 99.8	1	8.3	0.2 – 38.5
	Total	***145***	***87.7***	***83.1 – 93.3***	***18***	***11.0***	***6.7 – 16.9***

Table S9. Bait acceptance by owned and community (including strays or ownerless) dogs for the 3 bait types and 2 blister types; unknowns (ownership status) not included in data analysis.

		Owned			Community		
**Bait**	**Blister**	**n**	**%**	**95% CI**	**n**	**%**	**95% CI**
**Egg**	Hard	108	73.5	65.6 – 80.4	22	84.6	65.1 – 95.6
	Soft	89	84.8	76.4 – 91.0	13	72.2	46.5 – 90.3
	Subtotal	197	78.2	72.6 – 83.1	35	79.5	64.7 – 90.2
**Fish**	Hard	33	86.8	71.9 – 95.6	7	100.0	59.0 – 100
	Soft	27	75.0	54.1 – 84.6	5	83.3	35.9 – 99.6
	Subtotal	60	81.1	70.3 – 89.3	12	92.3	64.0 – 99.8
**Intestine**	Hard	27	84.4	67.2 – 94.7	8	100.0	63.1 – 100
	Soft	32	94.1	80.3 – 99.3	7	100.0	59.0 – 100
	Subtotal	59	89.4	79.4 – 95.6	15	100.0	78.2 – 100
**Subtotal**	Hard	168	77.4	71.3 – 82.8	37	90.2	76.9 – 97.3
	Soft	148	84.6	78.4 – 89.6	25	80.6	62.5 – 92.5
**Total**		**316**	**80.6**	**76.3 – 84.4**	**62**	**86.1**	**75.9 – 93.1**
